# Double Cystic Ducts Encountered During Laparoscopic Cholecystectomy in a Male Patient With Cholelithiasis: A Case Report

**DOI:** 10.7759/cureus.54814

**Published:** 2024-02-24

**Authors:** Aurangzeb Khan, Ghazanfar Khan, Shakeel Khan, Hamza Khan, Muhammad Asfandiyar

**Affiliations:** 1 Department of General Surgery, Mardan Medical Complex, Mardan, PAK; 2 Department of Radiology, Mardan Medical Complex, Mardan, PAK

**Keywords:** case report, laparoscopic cholecystectomy, h-type anomaly, cholelithiasis, double cystic duct

## Abstract

One of the most uncommon cystic duct abnormalities is double cystic ducts exiting a single gallbladder. Adequate knowledge of this anomaly should be kept in mind to avoid any surgical complications. We present a case of a 49-year-old Asian Pakistani male patient who had an elective laparoscopic cholecystectomy and was discovered to have two distinct cystic ducts leaving the gallbladder. On examination, there were no other clinically relevant signs except for mild tenderness in the right hypochondrium. Ultrasound of the abdomen and pelvis confirmed the diagnosis of cholelithiasis. Standard four-port laparoscopic cholecystectomy was done via the open Hasson technique under general anesthesia. After meticulous dissection of the Calot's triangle, double cystic ducts were discovered draining a single gallbladder. The gallbladder was retrieved after clipping and cutting the cystic ducts and cystic artery. To minimize the risk of complications, surgeons must be aware of the numerous anatomical variations that may exist.

## Introduction

Patients with abnormal biliary tree anatomy pose a greater risk of ductal damage and complications following surgery. Anomalies involving the development of the extra-hepatic biliary system exhibit incidence rates of 47% in the population [1]. Each year, more than 500,000 laparoscopic cholecystectomies are performed, highlighting the importance for surgeons to recognize the numerous potential anatomical anomalies, and reduce the likelihood of complications during these procedures [2]. Bile duct injury (BDI), with a 0.3% incidence rate, continues to be the most dreaded complication [3]. Cystic duct duplication with a single gallbladder is an extremely rare anomaly of the biliary tree, and the diagnosis of an accessory cystic duct is usually established intraoperatively [4].

In 1956, C. Steger reported the first case of cystic duct duplication [5]. Since then, only 17 cases of cystic duct duplication have been reported in the literature [6].

According to the literature, cystic duct variations increase the likelihood of ductal injury, necessitating open conversion and raising the risk of postoperative complications; however, they may go unrecognized during surgery and only be discovered subsequently through diagnostic investigations prompted by persistent biliary symptoms [7]. Notably, double cystic ducts are more commonly reported in the female population, with an incidence of 73% as compared to the male population [7]. In line with Consensus Surgical Case Report (SCARE) guidelines, we report a case of double cystic ducts with a single gallbladder in a male patient discovered intraoperatively during laparoscopic cholecystectomy [8]. Our case presents a rarity due to the patient's male gender, compounded by the absence of an intraoperative cholangiogram (IOC) during the procedure, which posed significant diagnostic and therapeutic challenges.

## Case presentation

A 49-year-old Asian-Pakistani married male patient, with a body mass index (BMI) of 33.3 kg/m², working as a government officer with a satisfactory economic status, presented to us in the outpatient department with chief complaints of recurrent biliary colic, associated with food intake, dull in nature, lasting approximately for half an hour for one month.

The patient had a negative past surgical history, no significant past medical history, no relevant drug history, and no known allergies to drugs or food. There was no significant family history of major disease and no history of smoking or alcohol consumption.

On examination, the patient was well-built and no pallor or jaundice was noted. Murphy’s sign was negative, but there was mild tenderness in the right hypochondrium. During bedside assessment, blood pressure was 130/78, pulse rate was 75 beats per minute (bpm), oxygen saturation was 98% on room air, and he was afebrile. Electrocardiography and echocardiography findings were normal.

Laboratory investigations, including complete blood count, liver function tests, renal function tests, serum electrolytes, and other blood investigations, were all within the normal range, as detailed in Table 1.

**Table 1:** Laboratory investigations pre and postoperatively WBC: white blood cells; Hb: hemoglobin; PLT: platelets; ALT: alanine transaminase; ALKP: alkaline phosphatase; BILIT: bilirubin total; AlbG: albumin; GGT: gamma-glutamyl transferase.

Investigation	Reference value	Result (pre-op)	Day 1 (post-op)	Day 10 (post-op)
WBC	4-11 10^3/ul	7.7	10.1	8.2
Hb	14-17 g/dl	15.0	14.8	15.2
PLT	150-400 10^3/ul	185.0	189.0	182.0
ALT	5-55 U/L	36	38	35
ALKP	40-150 U/L	112	125	115
BILIT	0.2-1.2 mg/dl	0.5	0.4	0.6
AlbG	3.5-5.2 g/dl	4.5	4.5	4.6
GGT	9-64 U/L	23	28	25
Serum creatinine	0.72-1.25 mg/dl	0.77		
Blood urea	10-50 mg/dl	29		
Sodium (Na)	135-148 mmol/L	137		
Potassium (k)	3.6-5.2 mmol/L	4.16		
Chloride (Cl)	98-108 mmol/L	98.1		

The viral profile, including hepatitis B surface antigen (HBsAg), human immunodeficiency virus (HIV), and hepatitis C virus (HCV), was negative and the urine routine examination was normal. The imaging study included abdominal ultrasonography, which showed a thick wall and edematous gallbladder with multiple gallstones; the largest one measured about 10 mm in diameter. All the intrahepatic and extrahepatic biliary channels were normal with a common bile duct of 3.5 mm in diameter.

**Figure 1:**
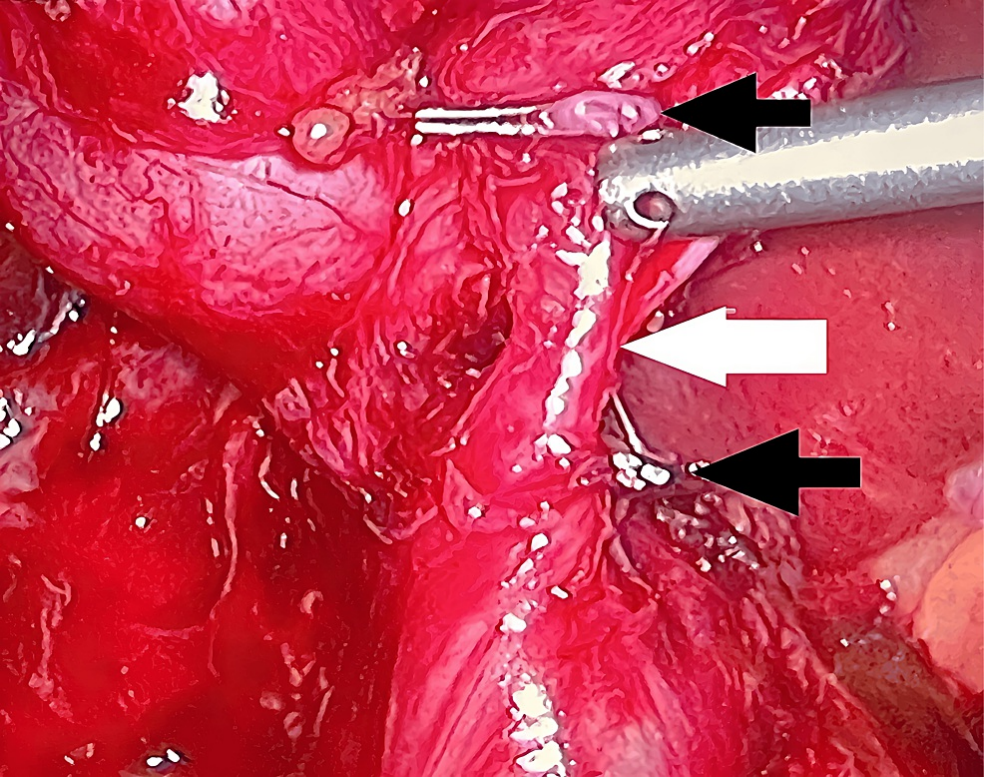
Two cystic ducts leaving the gallbladder (1) Accessory cystic duct clipped and cut (black arrows). (2) Intact cystic duct (white arrow).

The patient was counseled regarding the course of the disease. There was no lifestyle modification made preoperatively and the patient was not consuming any type of illicit or recreational drugs and drinks. A nil-by-mouth (NBM) status was maintained for eight hours preoperatively. The patient received an injection of tramadol 50 mg intravenously (IV) stat for pain relief, an injection of ceftriaxone 1 g IV stat for antibiotic prophylaxis, and an injection of omeprazole 40 mg IV stat preoperatively. The patient was transferred to the operation theater after filling out the preoperative checklist. Under the aseptic technique, the patient was draped and general anesthesia was given by a qualified anesthesiology team. After sedation, a standard four-port laparoscopic cholecystectomy was performed via the open Hasson technique. The visual umbilical port was 10 mm, the first working port was 10 mm sub-xiphisternum to the right, the second working port was 5 mm at the level of the gall bladder fundus, and the assistant port was 5 mm at the anterior axillary line. The patient was placed in reverse Trendelenburg position with a left tilt after establishing a pneumoperitoneum of 12 mmHg with carbon dioxide. After meticulous dissection of the Calot’s triangle above the level of Rouviere’s sulcus, double cystic ducts were accidentally identified leaving the gallbladder and entering the common bile duct separately (Figure 1). Each of the two cystic ducts and the cystic artery were clipped with two titanium Liga clips and cut independently.

The patient’s recovery from general anesthesia was uncomplicated. He was mobilized and was allowed a liquid diet six hours after surgery. He was observed in the surgical unit for 24 hours postoperatively and the hospital medications included injection of ketorolac 30 mg IV twice daily, injection of ceftriaxone 1 gm IV twice daily, injection of omeprazole 40 mg IV once daily, and infusion ringer lactate 500 ml IV twice daily. The patient was discharged home with oral medication, including a tablet of omeprazole 40 mg once daily for 14 days, a capsule of cefixime 400 mg once daily for five days, and a tablet of diclofenac (Na) 50 mg once daily for five days, along with a dietary instruction to avoid fatty and spicy foods and a two-week follow-up plan in the surgical outpatient department.

The patient faithfully adhered to the recommended diet and took his medications as prescribed. The patient's postoperative recovery was excellent. There were no intraoperative or postoperative complications observed.

## Discussion

Congenital anomalies of the extrahepatic biliary ducts have been observed to occur frequently with an incidence of 47% [1]. The duplication of the cystic duct is one of the rarest variations of the biliary tract. Embryologically, it is such that the gallbladder is initially a hollow organ, but it momentarily solidifies due to the growth of its epithelial lining. Vacuolation of the epithelium results in the formation of the final lumen, also known as recanalization. Duplicate bile ducts occur when the biliary system embryology deviates from the regular sequence or pattern of events. The development of accessory bile ducts is caused by the persistence of the fetal connection between the liver and gallbladder or the extrahepatic ductal system [9].

Duplication of the cystic duct is associated with a double gallbladder 80% of the time [2]. Rarely reports of a single gallbladder with multiple cystic ducts have appeared in the literature [4].

Only 27.3% of the reported cases of double cystic ducts were diagnosed preoperatively, whereas in 90.9% of the cases, the diagnosis of an accessory cystic duct was made intraoperatively. The enhanced visual effects of laparoscopic surgery and delicate dissection may be an explanation for this high rate of intraoperative diagnosis [4].

The duplicated cystic duct is classified into three types that include, H type (when they connect the right, left, or common hepatic duct) (as seen in our case), Y type (when they meet and form a single duct), and Trabecular type (when they enter the liver substance directly) [6]. There are biliary variations that may give the appearance of a double cystic duct, for example, segment VI's drainage into the cystic duct, the right posterior sector duct drains into the cystic duct, the right posterior sector duct's distal drainage into the gall bladder, and proximal right posterior sector duct drainage into the gall bladder's body [2].

In our case, two cystic ducts drained a single gallbladder and both of them entered separately into the common bile duct. Our case is unique because of the lack of availability of intraoperative cholangiogram, which posed major diagnostic and therapeutic challenges. For laparoscopic surgeons, this is not an uncommon situation to find themselves in, as many centers practicing elective laparoscopic cholecystectomy may not always be able to conduct standard intraoperative cholangiogram. However, after carefully examining the specimen, there was strong evidence to suggest that this was an accessory cystic duct, which allowed the procedure to be safely completed in the absence of an intraoperative cholangiogram. Additionally, having an H-type double cystic duct with a single gallbladder in a male patient is an extremely rare case and in cases with the H-shaped subtype, the possibility of injury to the bile duct and hepatic artery is high [10].

## Conclusions

Failure to recognize this rare condition might result in biliary tract injury, particularly transaction of the accessory cystic duct, resulting in bile leakage or fistula, duct ligation, or secondary cystic duct stricture. To avoid these complications, preoperative endoscopic retrograde cholangiopancreatography and intraoperative real-time cholangiogram should be performed. In the case of laparoscopic cholecystectomy, it is crucial to pay attention to such unusual congenital anatomy.
